# Resveratrol in benign prostatic hyperplasia: mechanistic insights and therapeutic perspectives

**DOI:** 10.3389/fimmu.2026.1874552

**Published:** 2026-08-25

**Authors:** Bailong Jiang, Tao Zhang, Maobin Yu, Yaodong You, Huawei Liu, Jinze Li, Peihai Zhang

**Affiliations:** 1 Department of Urology, Hospital of Chengdu University of Traditional Chinese Medicine, Sichuan, Chengdu, China; 2 Department of Urology, People’s Hospital of Deyang City, Chengdu University of Traditional Chinese Medicine, Deyang, China

**Keywords:** benign prostatic hyperplasia, fibrosis, inflammation, oxidative stress, resveratrol

## Abstract

Benign prostatic hyperplasia (BPH) is a common age-related disorder characterized by progressive prostatic enlargement and lower urinary tract symptoms, yet current medical therapies remain constrained by delayed onset, incomplete efficacy, and treatment-related adverse effects. Resveratrol (Res), a naturally occurring polyphenol, has emerged as a promising candidate for BPH intervention because its pharmacological profile aligns closely with the multifactorial biology of the disease. This review synthesizes current evidence showing that Res may counteract BPH progression through coordinated regulation of several interconnected processes, including abnormal prostate cell proliferation, apoptosis resistance, oxidative stress, chronic inflammation, fibrotic remodeling, and androgen-related signaling. Rather than acting on a single target, Res appears to reshape the pro-hyperplastic microenvironment at multiple levels, providing a mechanistic rationale for its therapeutic potential. However, the available evidence remains predominantly preclinical, and its clinical translation is still limited by poor oral bioavailability, extensive first-pass metabolism, uncertainty regarding the active molecular species in prostate tissue, and the lack of standardized formulations and robust clinical trials. Overall, Res represents a biologically plausible but not yet clinically established strategy for BPH management.

## 1 Introduction

Benign prostatic hyperplasia (BPH) is an age-associated, nonmalignant proliferative disorder of the prostate, histologically characterized by the expansion of epithelial and stromal compartments (1). Clinically, BPH often manifests as lower urinary tract symptoms (LUTS), including hesitancy, weak stream, urinary frequency, urgency, and nocturia. In advanced cases, it may lead to acute urinary retention, recurrent urinary tract infection, bladder dysfunction, or even renal impairment, substantially reducing quality of life and increasing healthcare utilization (1, 2). Both the prevalence of BPH and the burden of male LUTS increase substantially with advancing age, making BPH an increasingly important health concern in aging populations (3, 4). Current management strategies include pharmacological and surgical interventions. α1-Adrenergic receptor antagonists and 5α-reductase inhibitors remain the mainstays of medical therapy, whereas surgical treatment is generally reserved for patients with refractory symptoms, BPH-related complications, or clinically significant anatomical obstruction (2, 5). Although these approaches are effective in selected patients, their clinical utility may be limited by delayed therapeutic responses, incomplete symptom relief, treatment-related adverse effects, and suboptimal long-term adherence (5, 6). Surgical procedures can provide more immediate relief of obstruction but are associated with perioperative risks and potential functional complications (2, 5, 6). These limitations have stimulated growing interest in adjunctive strategies capable of targeting broader pathogenic mechanisms involved in BPH progression.

Resveratrol (Res), a naturally occurring polyphenolic compound, has attracted increasing attention because of its antioxidant, anti-inflammatory, antifibrotic, antiproliferative, and metabolic regulatory properties (7–9). Although BPH has long been regarded as an androgen-dependent condition, accumulating evidence indicates that its pathogenesis is multifactorial and involves chronic inflammation, oxidative stress, stromal remodeling, fibrosis, and metabolic disturbances associated with obesity and insulin resistance (1, 10–14). Notably, these pathogenic processes closely parallel the known pharmacological profile of Res, suggesting that this compound may have particular relevance in the context of BPH. Rather than acting through a single dominant target, Res may intervene in the pro-hyperplastic prostatic microenvironment at multiple levels by attenuating inflammatory and oxidative injury, limiting aberrant stromal and epithelial remodeling, and partially correcting endocrine–metabolic imbalance (15, 16). Against this background, interest in Res as a potential therapeutic candidate for BPH has increased in recent years. In this review, we summarize and critically appraise current evidence from BPH-related cellular models, animal studies, and limited human observations, with particular emphasis on the molecular mechanisms underlying its effects and on the major barriers that currently hinder its clinical translation.

## 2 Methods

### 2.1 Literature search

A structured literature search was conducted in PubMed, Web of Science Core Collection, and Scopus from database inception to July 16, 2026. The search strategy was developed around two principal concept domains: resveratrol and benign prostatic hyperplasia (BPH). Controlled vocabulary terms, free-text keywords, Boolean operators, and database-specific field tags were used as appropriate. No restriction on publication year was applied. The reference lists of potentially eligible articles and relevant reviews were also examined to identify additional studies that may not have been retrieved through the electronic database search. All retrieved records were compiled, and duplicate publications were removed before title and abstract screening.

### 2.2 Eligibility criteria

Studies were considered eligible when they met at least one of the following criteria (1): they directly investigated the effects of resveratrol in cellular models relevant to BPH (2); they evaluated resveratrol in animal models of BPH or prostate enlargement (3); they examined prostate- or BPH-related outcomes associated with resveratrol administration in human participants; or (4) they provided directly relevant mechanistic or translational evidence concerning the potential application of resveratrol in BPH.

Studies involving multicomponent preparations containing resveratrol or resveratrol analogues were included only when their findings were directly relevant to BPH and the contribution of resveratrol could be reasonably interpreted. Duplicate publications, conference abstracts lacking sufficient information, articles without available full text, and studies without a direct relationship to resveratrol and BPH were excluded. Studies focused exclusively on prostate cancer, prostatitis, or other prostate diseases were also excluded unless they provided mechanisms directly relevant to BPH.

## 3 Results

### 3.1 Literature search results

**Figure 1:**
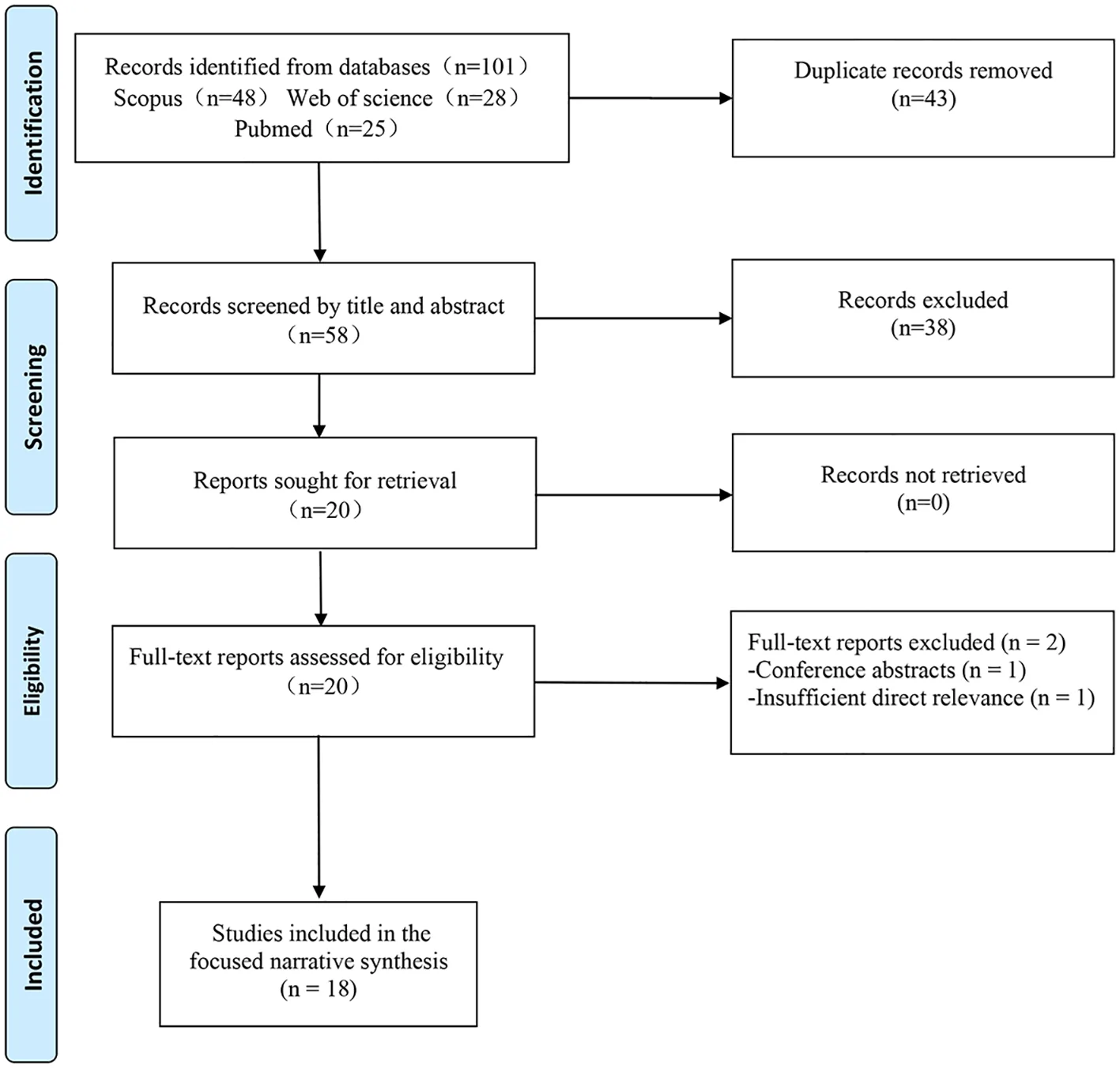
Flow diagram of the literature identification, screening, eligibility assessment, and study-selection process.

The database search yielded 101 records: 48 from Scopus, 28 from Web of Science, and 25 from PubMed. After removing 43 duplicates, 58 records underwent title and abstract screening, and 38 were excluded. The full texts of the remaining 20 articles were assessed for eligibility. Two articles were excluded because one was a conference abstract with insufficient information and the other lacked sufficient direct relevance to resveratrol and BPH. Ultimately, 18 studies were included in the focused narrative synthesis. The study selection process is presented in **Figure 1**.

## 4 Pharmacological properties and translational relevance of Res

### 4.1 Chemical structure and natural sources

Res is a naturally occurring non-flavonoid polyphenol with a characteristic stilbene backbone consisting of two phenyl rings linked by an ethylene bridge and substituted with hydroxyl groups. Res exists as cis- and trans-isomers, among which trans-Res is more abundant in nature, structurally more stable, and generally recognized as the predominant bioactive form (7, 9, 17). As a phytoalexin, Res is synthesized by plants in response to pathogen infection, ultraviolet irradiation, and other environmental stresses, thereby contributing to host defense (8, 9, 17, 18). Natural sources of Res include grape skins, red wine, peanuts, berries, and the traditional Chinese medicinal herb Polygonum cuspidatum (7, 9). Given its relative abundance and established medicinal use, Polygonum cuspidatum has become one of the major botanical sources for the extraction and production of Res (7, 9, 18).

### 4.2 Bioavailability and prostate tissue exposure

Orally administered Res is relatively well absorbed from the gastrointestinal tract; however, the systemic bioavailability of the unconjugated parent compound is extremely low because of extensive first-pass metabolism in the intestine and liver. Rapid sulfation and glucuronidation result in circulating profiles dominated by conjugated metabolites rather than free Res (19–21). For an organ-directed indication such as BPH, plasma concentrations of unconjugated Res alone may therefore provide an incomplete estimate of pharmacologically relevant exposure. In a window-of-opportunity study involving men undergoing prostate biopsy, daily administration of 5 mg or 1 g Res for 7–14 days resulted in detectable Res-derived compounds in prostate tissue. However, unconjugated Res was undetectable, whereas sulfate and glucuronide conjugates predominated (22). These metabolites exhibited limited cellular uptake and did not consistently reproduce the direct antiproliferative effects observed with micromolar concentrations of unconjugated Res *in vitro*. Moreover, current human prostate data provide no convincing evidence of substantial local deconjugation, and it remains uncertain whether these conjugates possess meaningful intrinsic activity or act as an effective local reservoir of parent Res. Although this study was not conducted specifically in patients with BPH, it demonstrates that prostate exposure following oral administration is dominated by conjugated metabolites rather than unconjugated Res. Accordingly, mechanistic findings obtained using high concentrations of unconjugated Res *in vitro* should be extrapolated to oral treatment with caution. Future studies should directly evaluate the cellular uptake, biological activity, and intracellular conversion of individual metabolites in BPH epithelial and stromal cells and integrate systemic pharmacokinetics, prostate tissue exposure, metabolite profiling, and pharmacodynamic endpoints.

### 4.3 Major pharmacological activities in BPH

Res exerts pleiotropic pharmacological activities that are highly relevant to the multifactorial pathobiology of BPH. Its principal effects include antioxidant and anti-inflammatory actions, regulation of cell-cycle progression and apoptosis, suppression of fibrotic remodeling, and improvement of metabolic homeostasis (7, 14, 16, 23).These effects are best viewed as network-level modulation rather than single-target inhibition. In redox regulation, Res can enhance antioxidant defenses through Nrf2/HO-1-related signaling while reducing excessive lipid peroxidation and inflammatory oxidant stress (9, 24). Its anti-inflammatory activity has been associated with modulation of NF-κB/STAT3 signaling and inflammasome-related pathways, which are also implicated in BPH progression (25–27). Res has been reported to modulate PI3K/AKT and ERK1/2 signaling, accompanied by alterations in cell-cycle regulators and apoptosis-associated proteins in BPH models (16, 28–31). Evidence from prostatic fibroblast and fibrosis-related models further suggests suppression of TGF-β1/Smad-dependent myofibroblast phenoconversion and extracellular matrix turnover (15, 32, 33). Collectively, these findings suggest that Res may target multiple pathological components of BPH through coordinated regulation of oxidative stress, inflammation, cell fate, and tissue remodeling. Therefore, Res represents a promising phytochemical candidate for BPH intervention, although further studies are required to clarify its precise molecular targets and translational potential.

## 5 Potential mechanisms of Res in BPH

Representative preclinical and clinical studies evaluating the effects of Res and Res-containing preparations in BPH-related contexts are summarized in **Table 1**. These studies have demonstrated that Res may exert beneficial effects through multiple pathological processes involved in BPH progression, including suppression of oxidative stress, attenuation of chronic inflammation, inhibition of fibrotic remodeling, and regulation of hormone-related signaling pathways (27, 28, 34–39).

**Table 1:** Animal studies and relevant human evidence for Res and Res-containing preparations in BPH.

Reference	Model/ population	Intervention	Key findings and interpretation
Xu et al., 2009 (34)	Hormone-induced BPH	Res 1, 5, 15 mg/kg/day, gavage, 2 weeks	Reductions in prostate index of 18.55% and 12.51% at 15 and 5 mg/kg/day, respectively. Improved the histopathological features of BPH in rats.
Chung et al., 2015 (28)	Hormone-induced BPH	Res 1 mg/kg/day, intraperitoneal injection, 4 weeks	Reduced prostate weight. Decreased cell proliferation and promoted apoptosis. Attenuated inflammatory responses.
Yang et al., 2017 (35)	Obesity-induced BPH	Res 50 mg/kg/day, gavage, 4 weeks	Reduced prostate weight and prostate index by approximately 31.5% and 31.8%, respectively. Suppressed prostatic hyperplasia in rats. Improved oxidative stress.
Calmasini et al., 2018 (36)	Obesity-induced BPH	Res 100 mg/kg/day, gavage, 2 weeks	Reduced prostate weight and epithelial hyperplasia. Decreased ROS production and gp91^phox expression. Improved insulin sensitivity and insulin-stimulated AKT phosphorylation.
Song et al., 2020 (37)	Hormone-induced BPH	Res-enriched peanut sprout extract 50, 100 mg/kg/day, gavage, 4 weeks	Induced cell-cycle arrest. Reduced tissue androgen levels. Decreased the expression of NF-κB and ERK1/2.
Jawad & Jasim., 2024 (38)	Hormone-induced BPH	Res 100 mg/kg/day, gavage, 4 weeks	Reduced the expression levels of inflammatory cytokines such as TNF-α 2.Attenuated oxidative stress.
Hata et al., 2024 (27)	Stromal-dominant BPH	Res 50, 100 mg/kg/day, intraperitoneal injection, 3 weeks	Reduced prostate weight and Ki-67 expression. Decreased NLRP3, IL-1β, and IL-18 expression. Supported involvement of the C5a–NLRP3 inflammasome pathway.
Kjær et al., 2015 (39)	Randomized, placebo-controlled, Men aged 30-60 years with MetS.	Res 150 or 1000 mg/day, 4 months	Reduced DHEA and DHEAS levels. No effect on prostate volume, PSA, testosterone, or DHT.

Res, resveratrol; BPH, benign prostatic hyperplasia; MetS, metabolic syndrome; DHEA, dehydroepiandrosterone; DHEAS, dehydroepiandrosterone sulfate; DHT, dihydrotestosterone; PSA, prostate-specific antigen; ROS, reactive oxygen species; AKT, protein kinase B; NF-κB, nuclear factor kappa B; ERK1/2, extracellular signal-regulated kinase 1/2; TNF-α, tumor necrosis factor-α; MDA, malondialdehyde; GPX, glutathione peroxidase; NLRP3, NOD-like receptor family pyrin domain-containing 3; IL-1β, interleukin-1β; IL-18, interleukin-18.

### 5.1 Proliferation-apoptosis imbalance

Disruption of the balance between cell proliferation and apoptosis contributes to the progressive accumulation of epithelial and stromal cells in BPH (10, 16, 31). In WPMY-1 cells, Res induced G0/G1-phase arrest, accompanied by increased expression of the cyclin-dependent kinase inhibitors p21 and p27 and reduced expression of cyclin D1, cyclin E, CDK2, and CDK4 (31). Res also reduced AKT and ERK1/2 phosphorylation and attenuated NF-κB DNA-binding activity. These findings indicate that suppression of proliferative signaling occurs in parallel with changes in the cell-cycle machinery. However, because pathway-specific rescue experiments were not performed, it remains uncertain whether the alterations in p21, p27, cyclins, and CDKs were directly mediated by AKT or ERK1/2 inhibition. A more recent integrated study likewise identified PI3K/AKT signaling as a prominent pathway associated with the effects of Res in BPH-related cells and experimentally demonstrated reduced PI3K and AKT phosphorylation, accompanied by decreased proliferation and increased apoptosis (16, 31). Given the established role of PI3K/AKT signaling in activating mTORC1, these observations further raise the possibility that mTOR-dependent growth signaling contributes to the antiproliferative effects of Res.

This possibility also provides a point of connection with the canonical metabolic targets of Res. mTORC1 promotes anabolic metabolism, protein synthesis, and cell-cycle progression, whereas AMPK can inhibit mTORC1 through TSC2- and Raptor-dependent mechanisms, thereby restraining cellular growth (40, 41). AMPK activation by Res may involve SIRT1 under certain experimental conditions, although SIRT1-independent activation has also been reported, indicating that SIRT1, AMPK, and mTOR are better regarded as an interconnected regulatory network than as a strictly linear cascade (42). Thus, the reductions in AKT phosphorylation and cell-cycle progression observed in Res-treated BPH stromal cells may be accompanied by attenuation of mTORC1-dependent growth signaling. Nevertheless, because SIRT1 activity, AMPK activation, and mTORC1 signaling were not directly examined in these BPH studies, this mechanistic connection remains hypothetical.

Beyond its effects on stromal proliferation, Res also induces pro-apoptotic responses in BPH epithelial cells. In BPH-1 cells, Res reduced cell viability and induced S-phase accumulation while increasing intracellular ROS, enhancing p38 MAPK phosphorylation, and decreasing FOXO3a protein expression (30). These changes were accompanied by reduced SOD2 and catalase expression, downregulation of Bcl-2 and Bcl-xL, and increased caspase-3 cleavage. Co-treatment with the ROS scavenger NAC or the p38 MAPK inhibitor SB203580 partially attenuated ROS accumulation, growth inhibition, and apoptosis, supporting the involvement of ROS-dependent p38 MAPK–FOXO3a signaling. Nevertheless, the molecular basis of the observed S-phase arrest remains unresolved.

Earlier *in vivo* findings showed that Res attenuated testosterone-induced prostatic enlargement, as reflected by reductions in the prostate index and improvements in histopathological features (34). In a subsequent hormone-induced BPH model, Res reduced prostate weight and proliferative activity, increased Bax expression, decreased Bcl-2 and Bcl-xL expression, and enhanced caspase-3 activation (28). Collectively, these findings suggest that Res may limit hyperplastic cell accumulation by regulating proliferative signaling, cell-cycle progression, and apoptosis-related pathways. Nevertheless, the evidence remains predominantly preclinical, and the relatively high concentrations used in several cell-based studies raise uncertainty about whether these mechanisms can be reproduced at pharmacologically achievable prostate tissue exposures.

### 5.2 Oxidative stress and inflammatory responses

Oxidative stress and chronic inflammation are increasingly regarded as interconnected components of the BPH microenvironment. Excessive ROS production and impaired antioxidant defense can induce oxidative DNA damage and activate redox-sensitive inflammatory pathways, whereas cytokines and inflammation-associated enzymes may further enhance ROS generation. This reciprocal interaction can sustain epithelial and stromal proliferation, disturb tissue repair, and promote extracellular matrix remodeling (12, 43). Supporting its pathological relevance, human BPH tissue exhibits increased oxidative DNA damage, with 8-hydroxy-2′-deoxyguanosine levels correlating with prostate weight (23). More recent clinical evidence also indicates that urinary inflammatory and oxidative stress biomarkers are associated with selected disease characteristics and treatment responses in patients with BPH (44). Collectively, these findings suggest that oxidative stress and inflammation constitute a mutually reinforcing pathological network rather than independent secondary responses, providing a rationale for interventions capable of modulating both processes.

BPH-specific studies indicate that Res can influence several components of this oxidative and inflammatory environment. In a hormone-induced BPH model, Res reduced prostate weight and proliferative activity while decreasing prostatic iNOS and COX-2 expression (28). Consistent with these findings, oral Res at 100 mg/kg for 28 days reduced prostatic TNF-α and malondialdehyde levels and increased glutathione peroxidase activity in a testosterone propionate-induced rat model, although Res was included as an active comparator to pterostilbene rather than as the primary intervention (38). In WPMY-1 stromal cells, Res attenuated NF-κB DNA-binding activity together with PI3K/AKT and ERK1/2 signaling, suggesting coordinated regulation of inflammatory and proliferative responses (31). More recently, Res reduced prostate weight and the expression of NLRP3, IL-1β, IL-18, and Ki-67 in a stromal-dominant BPH model, supporting the involvement of the C5a/NLRP3 inflammasome pathway (27). Evidence from an estradiol-induced chronic prostatitis model further showed reduced inflammatory infiltration and lower IL-6, IL-8, and TNF-α expression, although these findings provide only indirect support for BPH (45).

The reductions in NF-κB activity and NLRP3-associated inflammatory mediators observed in BPH models suggest a possible intersection with SIRT1- and AMPK/mTOR-related signaling (27, 31). SIRT1 can directly interact with RelA/p65 and suppress NF-κB-dependent transcription by deacetylating p65 at Lys310 (46). In LPS-stimulated macrophages, Res activated AMPK and inhibited NF-κB nuclear translocation and COX-2 expression, with pharmacological modulation of AMPK supporting its involvement in this anti-inflammatory response (47). In a non-prostatic model of chronic intermittent hypoxia, Res-associated AMPK activation was linked to suppression of the mTOR/TTP/NLRP3 mRNA regulatory pathway and reduced NLRP3 inflammasome activation (48). Other studies have shown that Res can also inhibit NLRP3 inflammasome activation by preserving mitochondrial integrity and enhancing autophagy, indicating that its effects on the inflammasome are unlikely to depend on a single pathway (49). Thus, SIRT1- and AMPK/mTOR-related signaling may contribute to the reductions in NF-κB activity and NLRP3-associated inflammation observed in BPH models. However, SIRT1 activity, p65 acetylation, AMPK activation, and mTOR signaling were not directly examined in these BPH studies, and this mechanistic connection remains hypothetical.

In obesity-associated BPH models, Res attenuated prostatic enlargement and oxidative injury. Yang et al. (35) reported reductions in prostate weight, oxidative stress, and histopathological hyperplasia following Res treatment. Similarly, Calmasini et al. (36) found that Res reduced prostate weight, epithelial hyperplasia, ROS production, and gp91^phox expression in obese mice, while improving insulin sensitivity and insulin-stimulated AKT phosphorylation. Conversely, in BPH-1 cells, Res increased intracellular ROS and activated p38 MAPK-FOXO3a signaling, thereby promoting apoptosis through reduced SOD2 and catalase expression (30). Thus, Res should not be viewed simply as a ROS scavenger; it may alleviate pathological oxidative stress *in vivo* while inducing pro-apoptotic redox signaling under specific cellular conditions. Further studies are required to define the relative contributions and causal relationships of NF-κB, NLRP3, and other redox-sensitive pathways in BPH.

### 5.3 Fibrotic remodeling

Fibrotic remodeling is increasingly recognized as a component of BPH pathology and is characterized by fibroblast activation, myofibroblast accumulation, extracellular matrix deposition, and reduced tissue compliance. TGF-β signaling is a major driver of this process by promoting myofibroblast differentiation and collagen production, which may increase prostatic stiffness and contribute to LUTS (14). Direct BPH-specific evidence for the antifibrotic effects of Res is derived primarily from cellular studies. Gharaee-Kermani et al. demonstrated that Res repressed and partially reversed TGF-β- or CXCL12-induced myofibroblast phenoconversion in primary and immortalized prostate fibroblasts, as indicated by reduced co-expression of COL1 and α-SMA (15). In BPH-1 epithelial and WPMY-1 stromal cells, Res counteracted TGF-β1-induced DIO3OS expression and modulated the downstream miR-656-3p/miR-485-5p–CTGF/ZEB1 network in a Smad-dependent context, thereby attenuating epithelial–mesenchymal transition and stromal cell proliferation (50). These findings support a potential effect of Res on epithelial–stromal remodeling, although the latter study did not directly assess extracellular matrix deposition or tissue-level fibrosis.

The modulation of TGF-β-related remodeling by Res may intersect with AMPK, mTOR, and SIRT1 signaling. In fibroblasts, TGF-β activates mTORC1 through a PI3K/AKT/TSC2-dependent mechanism, whereas mTORC2 contributes to TGF-β-induced morphological transformation and AKT phosphorylation (51). However, inhibition of mTORC1 did not directly suppress TGF-β-induced extracellular matrix protein expression in that model, suggesting that the contribution of mTOR may depend on the specific fibrotic endpoint. By contrast, AMPK activation has been shown to inhibit Smad3-dependent transcription, extracellular matrix production, and myofibroblast transdifferentiation (52). SIRT1 may also regulate TGF-β-responsive fibrotic signaling, although its effects appear to be tissue- and context-dependent: SIRT1 activation attenuated fibrotic responses in some experimental models but potentiated canonical TGF-β/Smad signaling in others (53, 54). Thus, AMPK/mTOR- and SIRT1-associated mechanisms may contribute to the effects of Res on TGF-β-related remodeling, but this connection has not been directly established in BPH epithelial or stromal cells.

Additional, albeit indirect, evidence is available from inflammation-associated prostatic fibrosis. In a chronic prostatitis rat model, Res reduced mast-cell activation, TGF-β/Wnt/β-catenin signaling, α-SMA expression, and collagen deposition while improving voiding function (55). A randomized placebo-controlled study in men with LUTS and inflammation-associated prostatic fibrosis reported improvements in NIH-CPSI and IPSS scores and reductions in leukocyte counts in expressed prostatic secretions after two months of Res supplementation (56). However, the clinical study did not directly assess histological fibrosis, extracellular matrix remodeling, or prostate stiffness. Thus, the available evidence suggests potential antifibrotic activity of Res in the prostate, but direct confirmation in conventional BPH models and objective clinical evidence of fibrosis regression remain lacking.

### 5.4 Androgen signaling

Androgen signaling remains a central component of BPH pathobiology. Within the prostate, testosterone is converted by 5α-reductase, particularly SRD5A2, into the more potent androgen dihydrotestosterone (DHT). DHT activates androgen receptor (AR) signaling in epithelial and stromal compartments, where compartment-specific transcriptional and paracrine effects contribute to prostate growth and epithelial–stromal interactions (1, 57). The clinical efficacy of 5α-reductase inhibitors further supports the importance of this pathway in BPH.

Evidence that Res directly modulates androgen signaling in BPH remains limited. In WPMY-1 stromal cells, Res reduced the expression of 5α-reductase, AR, and FGF-2 together with AKT and ERK1/2 phosphorylation (31). However, pathway-rescue experiments were not performed, and it remains unclear whether these changes reflect direct inhibition of androgen signaling or broader suppression of cell proliferation. A Res-enriched peanut sprout extract also reduced prostatic DHT and downregulated 5α-reductase, AR, and FGF expression in a testosterone-induced BPH model (37). Because the extract contained multiple bioactive constituents, these findings cannot be attributed solely to Res.

The concurrent changes in AR-related markers and AKT phosphorylation raise the possibility of crosstalk with SIRT1/AMPK/mTOR signaling. In prostate cancer cells, Res reduced AR protein stability and inhibited AR acetylation, DNA binding, and ligand-induced nuclear accumulation, thereby suppressing AR-dependent transcription (58, 59). SIRT1 has also been identified as a component of the corepressor complex recruited by antagonist-bound AR and is required for the repression of androgen-responsive genes under this specific experimental condition (60). In addition, AMPK activation can reduce AR nuclear localization and transcriptional activity, whereas AR signaling may reciprocally influence AMPK activity (61). AR signaling also interacts with PI3K/AKT/mTOR pathways in prostate cancer cells: androgen stimulation can activate mTOR-dependent growth signaling, whereas inhibition of PI3K/AKT/mTOR may alter AR activity through reciprocal feedback mechanisms (62, 63). These findings provide a potential mechanistic context for the effects observed in Res-treated BPH stromal cells. However, these interactions have been characterized predominantly in malignant prostate cells, and direct evidence that SIRT1, AMPK, or mTOR mediates the effects of Res on AR signaling in benign prostatic tissue is currently lacking.

**Figure 2:**
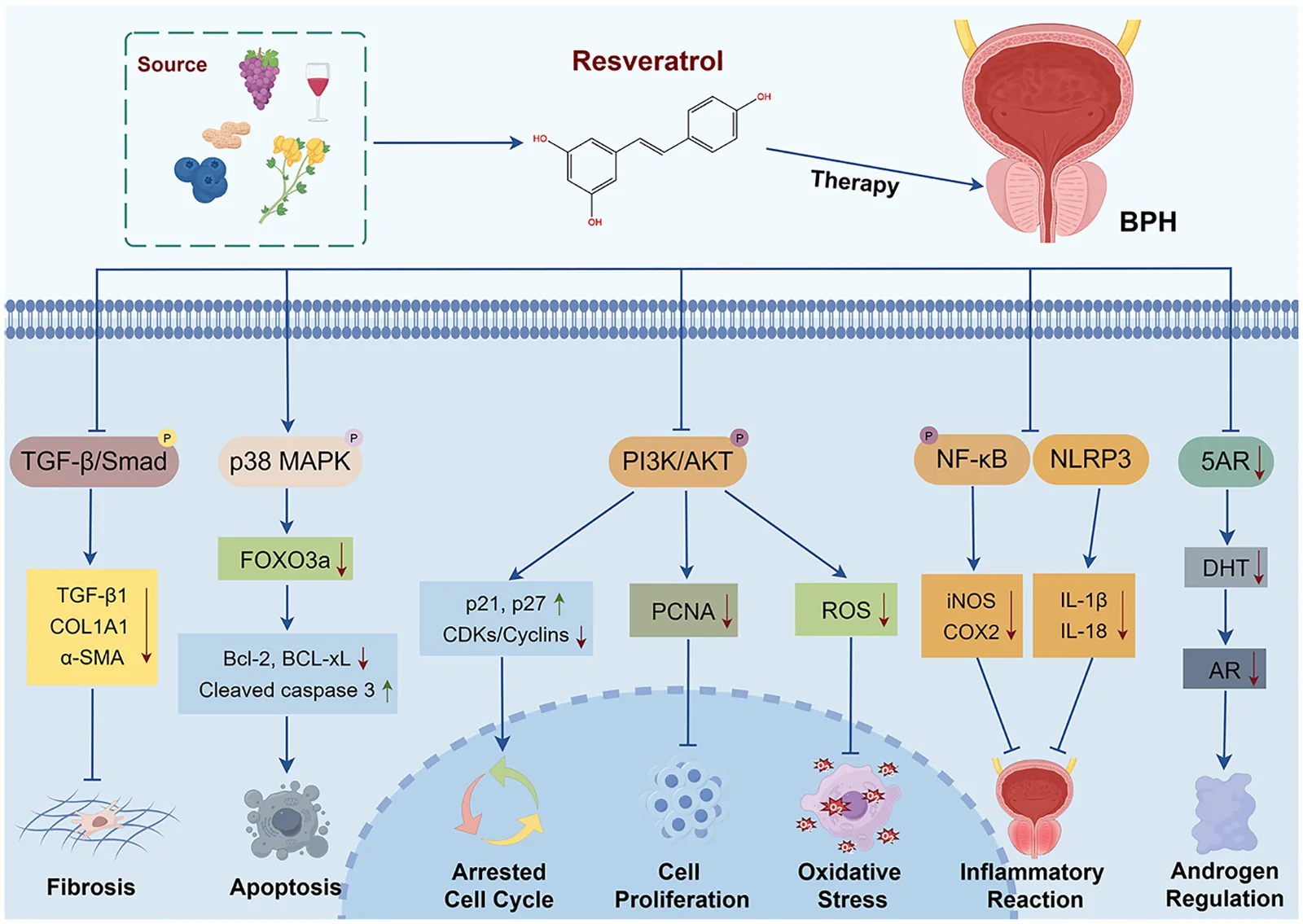
Proposed mechanisms of resveratrol in BPH. In BPH-related models, resveratrol may attenuate fibrotic remodeling by suppressing TGF-β/Smad signaling and reducing TGF-β1, COL1A1, and α-SMA expression. It may regulate cell-cycle progression and proliferation through inhibition of PI3K/AKT signaling, accompanied by increased p21 and p27 expression and decreased cyclins, CDKs, and PCNA. Resveratrol may also modulate p38 MAPK/FOXO3a signaling and apoptosis-related proteins. In addition, suppression of NF-κB and NLRP3 signaling may reduce iNOS, COX-2, IL-1β, and IL-18 expression, thereby attenuating inflammatory responses. Resveratrol may further influence androgen-dependent prostatic growth by reducing DHT- and AR-related signaling. Arrows indicate activation or downstream regulation, blunt-ended lines indicate inhibition, and upward and downward arrows indicate increased and decreased expression, respectively.

In a randomized trial involving men with metabolic syndrome rather than patients with BPH, four months of Res supplementation reduced several circulating androgen precursors but did not alter testosterone, DHT, PSA, or prostate volume (39). Thus, current evidence suggests that Res may influence androgen-related signaling in preclinical BPH models, but does not establish a clinically relevant effect on the DHT–AR axis. Further studies using purified Res, pharmacologically relevant exposure levels, and direct measurements of intraprostatic DHT, SRD5A activity, and AR transcriptional activity are required. The proposed mechanisms by which Res may modulate BPH progression are summarized in **Figure 2**.

## 6 Translational challenges and future directions

Although preclinical studies suggest that Res may modulate several pathological processes involved in BPH, its clinical relevance remains uncertain because the available models reproduce only selected components of this heterogeneous disease. Androgen-induced models primarily reflect androgen-dependent glandular enlargement, whereas obesity-associated models emphasize metabolic dysfunction, insulin resistance, and oxidative stress. Stromal-dominant models provide additional evidence concerning inflammasome-associated proliferation, while inflammation-related prostate models support a potential effect on fibrotic remodeling. Accordingly, a comparatively stronger rationale exists for prioritizing Res research in BPH associated with metabolic syndrome or prominent inflammatory and oxidative stress features. Androgen-dependent glandular enlargement may also be responsive, whereas BPH with prominent fibrotic features remains a more speculative indication because objective antifibrotic efficacy has not been demonstrated in conventional BPH models or phenotype-stratified clinical studies.

Poor and variable bioavailability represents another major barrier. Micronized formulations, prodrugs, and nanoparticle- or lipid-based delivery systems, including proliposomal formulations, may improve solubility, stability, intestinal absorption, or systemic exposure (64–69). However, greater plasma exposure cannot be assumed to increase pharmacologically active unconjugated Res in the prostate, where sulfate and glucuronide metabolites predominate following oral administration (22). Moreover, prostate-selective delivery, long-term carrier safety, manufacturing scalability, and regulatory feasibility remain unresolved. Pterostilbene exhibits more favorable oral pharmacokinetics than Res in rodents, but it is a distinct analog rather than a bioequivalent formulation, and its efficacy and prostate exposure in BPH require independent evaluation (70). Future development should therefore prioritize standardized and scalable formulations accompanied by direct measurement of parent Res and its major metabolites in plasma and prostate tissue.

Safety and drug–drug interactions are particularly relevant because BPH predominantly affects older men with frequent polypharmacy. High-dose Res has been associated mainly with gastrointestinal adverse effects (71). Experimental evidence of antiplatelet activity raises the possibility of additive effects with anticoagulant or antiplatelet agents, although the clinical bleeding risk remains undefined (72, 73). Reported effects on blood pressure have been inconsistent; nevertheless, additive hypotension remains a theoretical concern when Res is combined with α1-adrenergic blockers or other antihypertensive drugs (74, 75). Res at pharmacological doses has also been reported to modulate the phenotypic activities of CYP3A4, CYP2D6, CYP2C9, and CYP1A2, although the clinical significance of these changes for individual co-administered drugs remains uncertain (76). Endocrine effects should also be considered because high-dose Res reduced circulating DHEA and DHEAS in middle-aged men without significantly altering testosterone, DHT, PSA, or prostate volume (39). Future trials should therefore include medication reconciliation and prospective monitoring of gastrointestinal tolerability, bleeding, orthostatic blood pressure, hepatic and renal function, and endocrine parameters.

A staged clinical development strategy would be appropriate, beginning with pharmacokinetic and dose-finding studies to identify a standardized formulation with reproducible exposure and acceptable safety. A subsequent randomized, double-blind, placebo-controlled trial could evaluate resveratrol over 6-12 months in men with moderate LUTS and objectively documented prostate enlargement. Resveratrol could be assessed either as monotherapy in treatment-naïve patients or as an adjunct to stable α1-adrenergic blocker or 5α-reductase inhibitor therapy, with these therapeutic questions analyzed separately. The primary endpoint could be the change in International Prostate Symptom Score, with disease-specific quality of life, maximum urinary flow rate, post-void residual urine volume, prostate volume, and safety as secondary outcomes (77). Stratification by baseline prostate volume, metabolic syndrome, and inflammatory or oxidative stress status may help identify potentially responsive phenotypes. Pharmacokinetic and exploratory biomarker assessments could include parent resveratrol and its major metabolites, metabolic and inflammatory markers, and androgen-related hormones. Although no study has directly evaluated whether Res modifies prostate stiffness, shear-wave elastography may provide a noninvasive exploratory measure of fibrotic remodeling in future BPH trials. Fibrosis- and proliferation-related tissue markers, including α-SMA, collagen I, TGF-β1, Ki-67, and NLRP3, could also be examined in participants undergoing clinically indicated prostate surgery. Longer-term follow-up would be required to determine whether resveratrol affects acute urinary retention, treatment escalation, or BPH-related surgery (78, 79).

## 7 Conclusion

Res is a biologically plausible candidate for BPH intervention because its pharmacological actions overlap with several pathological processes involved in disease progression. Current evidence suggests that Res may regulate cell proliferation through signaling pathways such as PI3K/AKT, promote apoptosis through pathways such as p38 MAPK–FOXO3a, suppress inflammation through pathways such as NF-κB and NLRP3, attenuate fibrosis through pathways such as TGF-β/Smad, and influence androgen signaling. However, these findings are derived predominantly from cell-based and animal studies, and the causal relationships among the proposed mechanisms remain incompletely defined. Clinical evidence is limited and largely indirect, while poor bioavailability, extensive metabolism, heterogeneous dosing regimens, and uncertain prostate tissue exposure remain major barriers to translation. Future studies should validate key mechanisms in standardized BPH models, identify the pharmacologically active Res-related species in prostate tissue, optimize delivery strategies, and conduct adequately powered BPH-specific trials that include symptomatic, structural, functional, and biomarker outcomes. Therefore, Res should currently be regarded as a promising mechanistic lead rather than an established treatment for BPH.
